# Protective Effect of Polysaccharides Isolated from *Sargassum horneri* against H_2_O_2_-Induced Oxidative Stress Both In Vitro, in Vero Cells, and In Vivo in Zebrafish

**DOI:** 10.3390/biology13090651

**Published:** 2024-08-23

**Authors:** Shuangyan Wei, Li Wang, Jia Yang, Ruihang Xu, Rui Jia, Peimin He

**Affiliations:** 1 College of Oceanography and Ecological Science, Shanghai Ocean University, Shanghai 201306, China; wsy97221@163.com (S.W.); orange709wl@163.com (L.W.); yangjia0808@163.com (J.Y.); xrh_cn@163.com (R.X.); 2 Marine Biomedical Science and Technology Innovation Platform of Lingang Special Area, Shanghai 201306, China

**Keywords:** *Sargassum horneri*, polysaccharides, oxidative stress, Vero cell, zebrafish

## Abstract

**Simple Summary:**

The environment within which human beings exist is progressively becoming more complex. A multitude of external factors prompt the body to produce excessive amounts of reactive oxygen species (ROS), thereby leading to heightened levels of oxidative stress, which is detrimental to health. In recent years, researchers have discerned the potential of secondary metabolites derived from brown algae in combating diseases. Additionally, *Sargassum horneri* has witnessed a large-scale outbreak in China, exerting a severe negative influence on the local ecology and economic development. We extracted polysaccharides (SHPs) from *S. horneri* and discovered that SHPs possess outstanding antioxidant properties and can be utilized as a natural antioxidant. Our work can alleviate the environmental stress caused by algae and achieve the utilization of algae resources, which hold considerable social value and significance.

**Abstract:**

The extensive outbreak of *Sargassum horneri* in China has not merely imposed a severe threat to the ecological environment and human life in coastal waters but also impeded the development of waterway transportation and the local economy. Consequently, we isolated polysaccharides from *S. horneri*, designated as SHP, and evaluated the antioxidant activity of SHP both in vitro and in vivo by investigating the effect of SHP on H_2_O_2_-induced African green monkey kidney cells (Vero cells) and zebrafish. The results demonstrated that SHP can enhance the activities of superoxide dismutase, catalase, and glutathione peroxidase in zebrafish. It also effectively inhibits micro malondialdehyde and ROS levels in Vero cells and zebrafish to mitigate the oxidative damage caused by H_2_O_2_, thereby achieving the protective effect of SHP on Vero cells and zebrafish. In conclusion, SHP holds the potential as a natural antioxidant. SHP can be contemplated for utilization as a natural antioxidant in the biomedical, cosmetic, and food industries, thereby alleviating the environmental stress caused by *S. horneri* and achieving resource utilization.

## 1. Introduction

With the swift advancement of industry, science, and technology, human beings inhabit an increasingly intricate environment. Reactive oxygen species (ROS) will escalate in response to external stimuli (including ultraviolet light, cigarette smoke, air pollution, etc.) [1]. Generally, ROS produced by normal metabolism can be eliminated by endogenous antioxidant systems. However, excessive environmental stress will result in the abnormal generation of ROS, thereby causing oxidative stress [1,2]. Oxidative stress, a series of reactions caused by an imbalance between reactive oxygen species and the body’s antioxidant system, is extensively implicated in the progression of numerous chronic diseases such as neurodegenerative disorders, diabetes, cancer, inflammation, cardiovascular diseases, aging, and many geriatric diseases [3,4]. Elevated levels of superoxide dismutase (SOD), catalase (CAT), and glutathione peroxidase (GSH-PX) can down-regulate the production of ROS. Antioxidants can enhance the activity of these enzymes, not only possessing a potent free radical scavenging capacity but also regulating signaling pathways like MAPK, NF-κB, and Nrf2, lowering the level of ROS production, protecting cells from oxidative stress damage, and thereby mitigating the related diseases caused by oxidative stress [5,6]. Natural antioxidants are safer, more moderate, and have fewer side effects compared to chemically synthesized antioxidants [7]. Hence, natural bioactive substances with robust ROS scavenging ability will be ideal therapeutics for oxidative stress diseases.

Polysaccharides isolated from seaweed have been reported to possess numerous health benefits, including antioxidant, UV protection, liver protection, anti-inflammatory, antibacterial, and anticancer activities [8,9,10,11,12,13,14,15]. Furthermore, the polysaccharide content in seaweed is particularly abundant, which constitutes one of the most crucial bioactive substances in seaweed [16,17]. The research suggests that extracts such as polysaccharides in *Fucus vesiculosus* and *Turbinaria conoides* exhibit antioxidant properties and that they could be employed to prevent skin aging and diseases [18]. The polysaccharides isolated from *Ecklonia cava* demonstrated outstanding antioxidant activity in both in vivo and in vitro experiments [19]. The study indicated that the polysaccharides extracted from *Padina boryana* can significantly reduce oxidative stress in Vero cells and zebrafish [20]. The polysaccharides isolated from *Codium fragile* through enzyme-assisted extraction have been shown to inhibit H_2_O_2_-induced oxidative stress both in vitro and in vivo [8], which indicates that seaweed polysaccharides are highly promising natural antioxidants. Previous studies have unveiled the capacity of *Sargassum horneri* polysaccharides to scavenge free radicals, and the in vitro antioxidant effects of *S. horneri* polysaccharides have been evaluated at the cellular level [21,22,23,24]. Additionally, the anti-inflammatory [25,26,27,28], anti-tumor [29], immunomodulatory [30], whitening [24], and moisturizing effects [21,31] of *S. horneri* polysaccharides have been explored, suggesting a wide array of potential applications.

*S. horneri* is an edible brown alga, predominantly distributed in the warm temperate sea regions of China. It exhibits rapid growth and possesses a substantial biomass. Consequently, it often floats in patches on the sea surface, subsequently forming an extensive seaweed field, which is even referred to as the “forest in the sea” [32]. Widespread outbreaks of *S. horneri* have occurred in China, inflicting severe damage on tourism, aquaculture, and waterways, and even posing a threat to the ecological environment and human health [33,34,35]. Hence, it is highly significant to develop *S. horneri* polysaccharides as antioxidants, as this can not only reduce the environmental pressure of offshore waters but also achieve resource utilization. We chose to employ hydrogen peroxide (H_2_O_2_) to induce oxidative stress to investigate the antioxidant effect of the polysaccharide extract of *S. horneri* in Vero cells and zebrafish.

## 2. Materials and Methods

### 2.1. Chemicals and Regents

The African green monkey kidney cell (Vero) was purchased from Procell Life Science and Technology Co., LTD (Wuhan, China). The wild-type zebrafish was provided by the College of Fisheries and Life Sciences of Shanghai Ocean University. Minimum essential medium (MEM), dimethyl sulfoxide (DMSO), penicillin/streptomycin (P/S), and trypsin/EDTA were purchased from Nanjing BioChannel Biotechnology Co., LTD (Nanjing, China). Fetal bovine serum (FBS) was obtained from Gibco (Thermo Fisher, Mulgrave, VIC, Australia). 2′,7′-dichlorodihydrofurescin diacetate (DCFH-DA), cell counting kit-8 (CCK-8), and protein assay kit (BCA kit) were purchased from Beyotime Biotechnology Co., LTD (Shanghai, China). Catalase activity (CAT) assay kit, superoxide dismutase (SOD) assay kit, micro malondialdehyde (MDA) assay kit, and glutathione peroxidase (GSH-PX) assay kit were bought from Nanjing Jiancheng Bioengineering Institute (Nanjing, China). All other chemicals and reagents were of analytical grade.

### 2.2. Plant Material and Sample Preparation

*S. horneri* was collected in October 2020 from Gouqi Island, Zhejiang Province. The preparation of polysaccharides has been described in previous work [36]. The polysaccharide of *S. horneri* obtained was named SHP. Moreover, the SHP was characterized by FT-IR measurements within the frequency range of 500–4000 cm^−1^.

### 2.3. The Cell Cultures

The Vero cells were cultured in a medium comprising 10% FBS and 1% penicillin/streptomycin, and placed in a cell culture chamber with 5% CO_2_ and a temperature of 37 °C. Once the cells reached a growth density ranging from 80% to 90%, they were digested using 0.25% trypsin and re-culture. Subsequently, cells in the logarithmic phase were selected for the subsequent experiments.

### 2.4. The Assay for Cytotoxicity

Vero cells were seeded into 96-well plates at a density of 1 × 10^5^ cells mL^−1^. After 24 h of cell culture in the cell incubator, the cells were exposed to various concentrations of H_2_O_2_ (0.1, 0.2, 0.3, 0.4, 0.5, 0.6 mmol L^−1^) to establish the H_2_O_2_-induced damage model. Alternatively, Vero cells were treated with different concentrations of SHP (0, 25, 50, 75, 100, 150, 200 μg mL^−1^), and then the effect of SHP on the survival rate of Vero cells was determined. Alternatively, the same volume of SHP (25, 50, and 100 μg mL^−1^) was pre-treated for 1 h, followed by the treatment of Vero cells with H_2_O_2_ (0.4 mmol L^−1^), while the control group was treated with medium alone to determine the protective effect of SHP on the viability of Vero cells induced by H_2_O_2_. The cells were further cultured for another 24 h, and then CCK-8 solution was added, and the absorbance was measured at 450 nm (iBIO-RAD Mark, Vallejo, CA, USA).

### 2.5. Measurement of Intracellular ROS Levels

The DCFH-DA probe per se has no fluorescence. Once DCFH-DA enters cells, it will be oxidized by ROS and transformed into a green, fluorescent substance. Therefore, it is frequently employed to detect the level of ROS within cells [37]. Vero cells were seeded into 6-well plates at a cell density of 1×10^5^ cells/mL; the same volume of SHP (25, 50, and 100 μg mL^−1^) was pretreated for 1 h, and subsequently, the Vero cells were treated with H_2_O_2_ (0.4 mmol L^−1^), while the control group was treated merely with the medium. After 24 h incubation, DCFH-DA with a final concentration of 10 μg mL^−1^ was added, and the incubation was carried out for 30 min in the dark. The medium was cleaned twice. The cells were collected, the fluorescence intensity within the Vero cells was measured, and the observations were conducted under a fluorescence microscope (Nikon, Tokyo, Japan).

### 2.6. The Effect of SHP on Intracellular MDA Content and SOD Activity

When the body is stimulated by the external environment, ROS are abnormally produced. However, SOD can convert superoxide into hydrogen peroxide and oxygen, while maintaining ROS within the body within an appropriate range to reduce the damage caused by oxidative stress [38]. MDA is recognized as the ultimate decomposition product of lipid peroxidation. The concentration of MDA within a biological sample can be utilized as an indicator of the degree of damage sustained by the cell due to oxidative stress [39]. Briefly, Vero cells were seeded as described above. After a 24 h culture period, the medium was discarded, washed twice with PBS, and the supernatant was discarded after centrifugation. Subsequently, 200 μL of deionized water was added to the cells, and the cells were disrupted by the repeated freeze–thaw method. The contents of SOD and MDA in the cells were measured in accordance with the kit instructions.

### 2.7. Maintenance of Zebrafish

The maintenance of zebrafish was as described in one study with minor modifications [17]. Briefly, fifteen wild-type adult zebrafish were fed three times daily in a tank at 28 ± 0.5 °C, under a 14/10 h light/dark cycle. A pair of adult zebrafish were mated in spawning tanks, and healthy embryos were collected within 120 min. The use of zebrafish adheres to the document of the Animal Ethics Committee of Shanghai Ocean University (SHOUDW-2016-003).

### 2.8. The Survival Rate of Zebrafish Embryos

The survival rate of zebrafish embryos was determined in accordance with the method described by Kim et al. [40]. Briefly, 20 zebrafish embryos fertilized were selected, cultured to 7–9 h post-fertilization (hpf), and various concentrations of H_2_O_2_ (0, 1, 3, 5, 7, 9, 11, 13, 15 mmol L^−1^) were added to induce zebrafish embryos for the establishment of an oxidative stress model. The zebrafish embryos were placed in a 28 °C incubator for further culturing for 96 h, while the survival rate and development of zebrafish embryos were recorded. A control group (culture medium), an induction group (H_2_O_2_), and SHP groups with different doses (25, 50, and 100 μg mL^−1^) were established to assess the protective effect of SHP on H_2_O_2_-induced oxidative stress of zebrafish embryos.

### 2.9. Measurement of Oxidative Stress-Related Indicators (SOD, CAT, GSH-PX, and MDA) in Zebrafish

The activities of SOD, CAT, and GSH-PX constitute a primary antioxidant defense system, which plays a crucial and fundamental role in the overall defense mechanisms and strategies within biological systems [41]. The control group (medium), induction group (H_2_O_2_), and SHP groups (25, 50, and 100 μg mL^−1^) were established to treat zebrafish embryos. Several zebrafish that developed to 96 hpf were collected and placed into a 1.5 mL centrifuge tube, and 2 to 3 magnetic beads were added to prepare a 10% tissue homogenate, followed by centrifugation to obtain the supernatant. The SOD activity, CAT activity, GSH-PX content, and MDA content were measured in accordance with the kit instructions.

### 2.10. Determination of ROS in Zebrafish

Zebrafish embryos were treated in accordance with the above description. Several zebrafish that had developed to 96 hpf were randomly selected. Subsequently, DCFH-DA was diluted in the embryo culture medium (with a final concentration of 10 μmol L^−1^), incubated for 30 min in the dark, and washed three times to eliminate DCFH-DA that had not entered the zebrafish body. The fluorescence microscope was utilized for observation and photography, and the fluorescence intensity in zebrafish was quantified (using Image J software V 1.8.0).

### 2.11. Statistical Analysis

The data are expressed as the mean ± standard error (S.E.), and the ANOVA test was employed for statistically comparing the mean values (with SPSS 26.0 statistical software). All experiments were performed in triplicate.

## 3. Results

### 3.1. FTIR Spectrum of S. horneri Polysaccharide (SHP)

The extraction and structural characterization of SHP have been elaborated in detail in our previous study [36]. The FTIR of SHP exhibited the presence of characteristic peaks specific to polysaccharides at 3432 cm^−1^, 2938 cm^−1^, 1625 cm^−1^, 1200 cm^−1^, and 842 cm^−1^ and indicated the presence of sulfate, pyranoside, α-glycosidic bond, and other features. In summary, the results demonstrate that SHP exhibits the characteristic features of sulfate polysaccharides.

### 3.2. Determination of the H_2_O_2_ Damage Model

**Figure 1:**
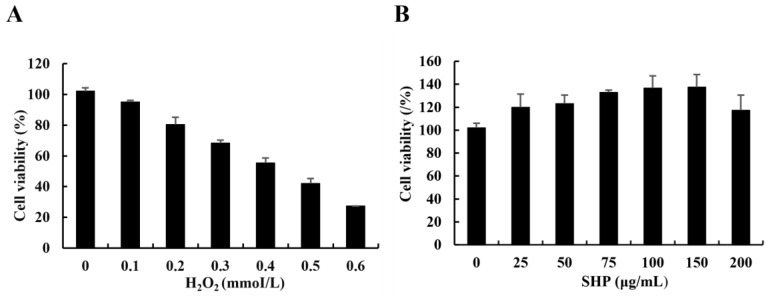
Vero cytotoxicity assay. (**A**) The effect of H_2_O_2_ on Vero cell viability. (**B**) The effect of SHP on Vero cell viability. The experiments were performed in triplicate and data are expressed as mean ± SE.

Currently, H_2_O_2_ has been extensively employed as an inducer in the assessment of antioxidant studies. Thus, in this study, Vero cells were exposed to diverse concentrations of H_2_O_2_ to construct a model of oxidative damage. The activity of Vero cells declined as the concentration of H_2_O_2_ increased (Figure 1A). In comparison with the control group, when the concentration of H_2_O_2_ was 0.4 mmol/L, the activity of Vero cells was 55.48%, which was approximately the lethal concentration 50 (LC_50_). Consequently, the H_2_O_2_-induced concentration of Vero cells was 0.4 mmol/L. Our results demonstrate that at SHP concentrations (25–200 μg mL^−1^), Vero cells were more than 90% active, indicating that SHP had no significant toxicity to Vero cells (Figure 1B). Therefore, three doses of 25, 50, and 100 μg/mL were chosen in the subsequent experiment to examine the antioxidant capacity of SHP in Vero cells.

### 3.3. The Effect of SHP against Oxidative Stress Induced by H_2_O_2_ in Vero Cells

**Figure 2:**
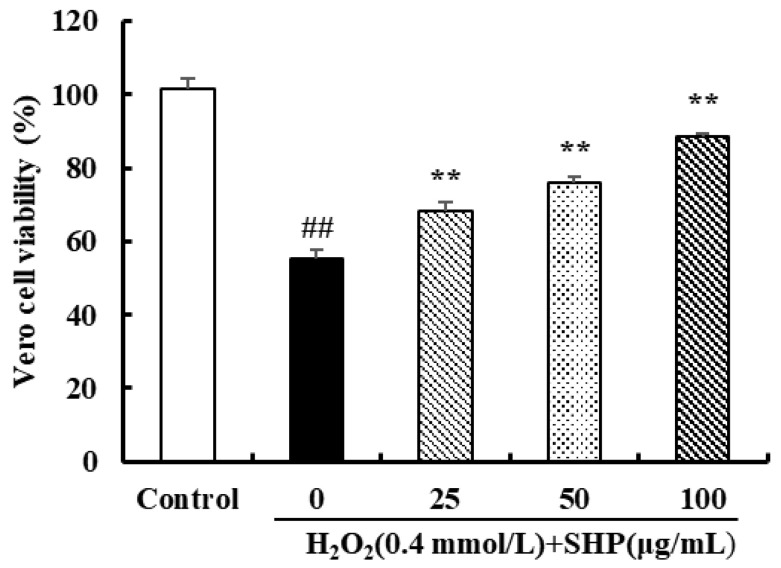
The effect of SHP on H_2_O_2_-induced oxidative damage in Vero cells. ^##^ *p* < 0.01 vs. control group; ** *p* < 0.01 vs. H_2_O_2_ injury group.

In contrast to the control group, Vero cells treated with H_2_O_2_ (0.4 mmol L^−1^) exhibited a significant decrease in cell viability, reaching merely 55.09% (*p* < 0.01) (Figure 2). However, in reality, the cell viability increased following treatment with SHP at concentrations of 25, 50, and 100 μg mL^−1^ in a dose-dependent manner. After treatment with 100 μg mL^−1^ SHP, the activity of Vero cells was restored to 88.62% (*p* < 0.01) (Figure 2). The results showed that SHP had a protective effect on H_2_O_2_-induced injury on Vero cells.

### 3.4. The Scavenging Effect of SHP on ROS in Vero Cells

**Figure 3:**
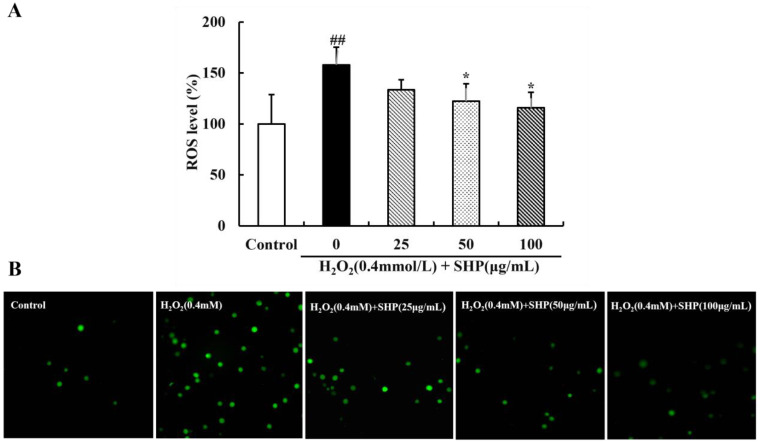
The effect of SHP on ROS generation in Vero cells induced by H_2_O_2_. (**A**) Results of the determination of fluorescence intensity; (**B**) fluorescence images. ^##^ *p* < 0.01 vs. control group; * *p* < 0.05 vs. H_2_O_2_ injury group.

Excessive levels of ROS can disrupt redox homeostasis and damage cell macromolecular structures such as cell membranes, proteins, and lipids, ultimately leading to apoptosis or cell death. Thus, it is of significance to maintain healthy ROS levels within cells [42]. The effects of SHP on ROS levels in Vero cells are shown in Figure 3. Compared with the control group, the fluorescence intensity of H_2_O_2_-induced Vero cells was significantly increased by 158.06% (*p* < 0.01). However, when the concentration of SHP was increased to 100 μg mL^−1^, the ROS level in Vero cells decreased to 116.01% (*p* < 0.05) (Figure 3). Meanwhile, the fluorescence microscopy images provide additional corroboration of this result (Figure 3). The aforementioned results demonstrate that SHP can effectively remove intracellular ROS to alleviate oxidative stress damage in Vero cells.

### 3.5. Effects of SHP on Intracellular MDA Content and SOD Activity

The SOD activity and MDA content in Vero cells are presented in Table 1. In contrast to the control group, the SOD activity of Vero cells was significantly decreased and the MDA content was significantly increased due to H_2_O_2_ induction (*p* < 0.01), indicating a high level of oxidative stress state. However, Vero cells treated with SHP (25, 50, and 100 μg mL^−1^) showed a significant increase in SOD activity compared to the induction group, while MDA levels were significantly reduced and returned to normal levels (*p* < 0.05 or *p* < 0.01). It is proved that SHP can reduce H_2_O_2_-induced oxidative damage in Vero cells.

### 3.6. Effects of H_2_O_2_ on Survival Rate and Hatching Rate of Zebrafish

**Figure 4:**
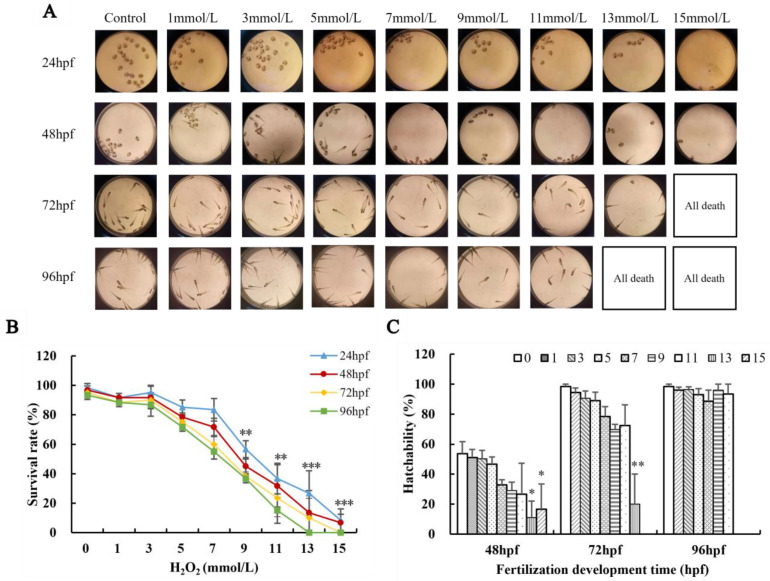
The effect of H_2_O_2_ on the survival and development of zebrafish. (**A**) Morphological growth and development diagram of zebrafish (×1.8 times); (**B**) survival rate of zebrafish; (**C**) hatching rate of zebrafish. Compared with the control group, * *p* < 0.05, ** *p* < 0.01, *** *p* < 0.001.

The survival rate and hatching rate of zebrafish are affected by the concentration of H_2_O_2_ (Figure 4). To establish an inflammation model of zebrafish, it is necessary to determine a safe dose of H_2_O_2_. A low concentration of H_2_O_2_ has little effect on the survival rate of zebrafish; however, the survival rate gradually decreases with the increase in time (Figure 4B). When the concentration of H_2_O_2_ exceeds 7 mmol L^−1^, the survival rate of zebrafish is 55% at 96 hpf, approximately half of the mortality rate (*p* < 0.01 or *p* < 0.001) (Figure 4). All zebrafish treated with H_2_O_2_ (13 mmol L^−1^) died at 96 hpf, and those with 15 mmol/L H_2_O_2_ all died starting at 72 hpf (*p* < 0.01 or *p* < 0.001) (Figure 4). The hatching rate of zebrafish also decreases with the increase in H_2_O_2_ concentration (Figure 4C). Thus, 7 mmol L^−1^ was chosen as the H_2_O_2_-induced concentration for further experiments.

### 3.7. Effects of H_2_O_2_ on the Growth and Development of Zebrafish

**Figure 5:**
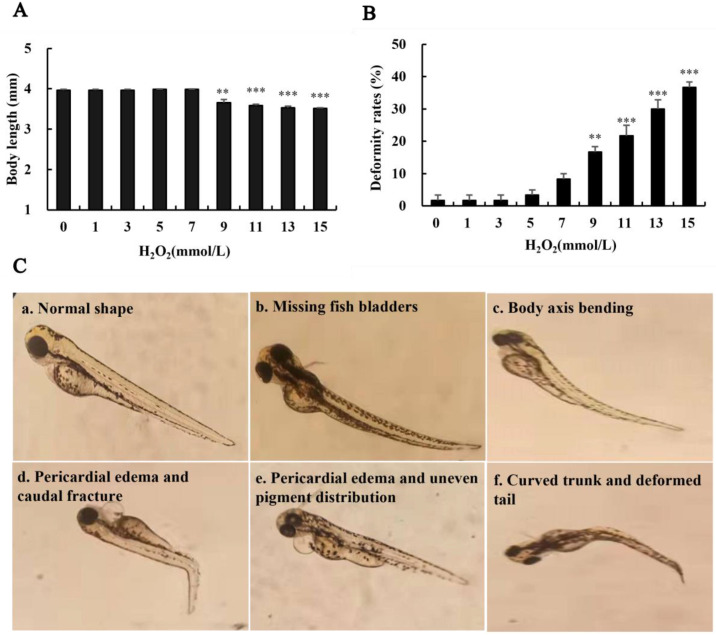
The effect of H_2_O_2_ on the morphological development of zebrafish (×4 times). (**A**) The body length of zebrafish; (**B**) deformity rates; (**C**) abnormal morphology of zebrafish (×4 times). Compared with the control group, ** *p* < 0.01, *** *p* < 0.001.

High levels of H_2_O_2_ can exert an impact on the normal development of zebrafish, resulting in certain deformities (Figure 5). The body length of zebrafish was negatively correlated with an H_2_O_2_ concentration above 7 mmol L^−1^, while the malformation rate was positively correlated with H_2_O_2_ (*p* < 0.01 or *p* < 0.001) (Figure 5). The morphology of zebrafish from fertilization to adult stage was observed under the microscope (Figure 5). It was found that the zebrafish in the control group showed normal body development and a uniform pigment distribution, whereas those treated with H_2_O_2_ showed various deformities (Figure 5C).

### 3.8. Effects of SHP on H_2_O_2_-Induced Zebrafish

**Figure 6:**
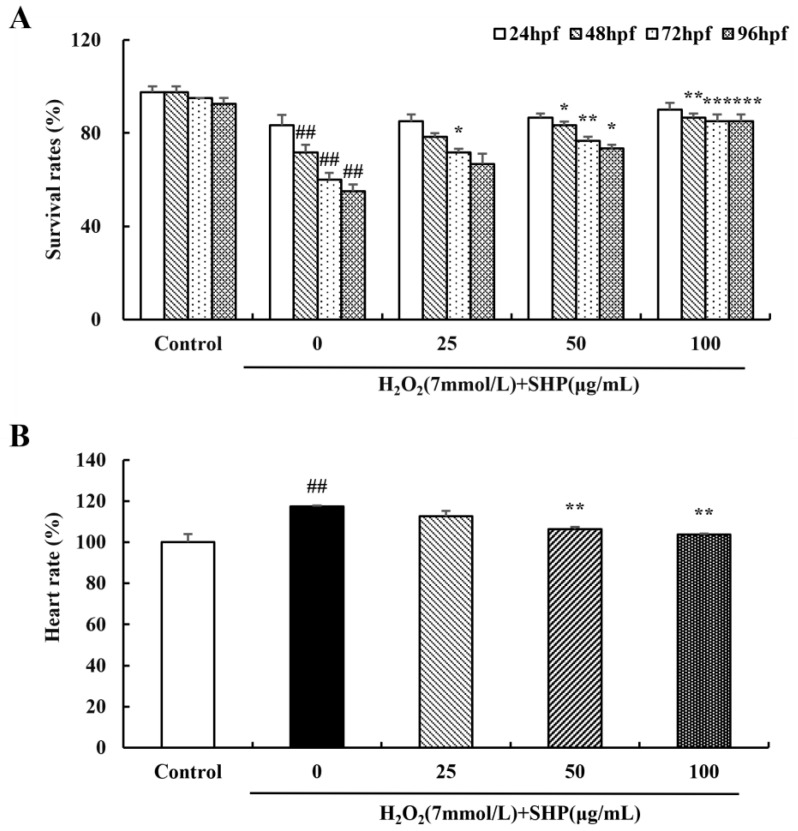
The effect of SHP on the survival and development of zebrafish embryos induced by H_2_O_2_ (**A**) The survival rate of zebrafish; (**B**) the heart rate of zebrafish. ^##^ *p* < 0.01 vs. control group; * *p* < 0.05, ** *p* < 0.01, *** *p* < 0.001 vs. H_2_O_2_ model group.

We determined the survival rate of zebrafish embryos to assess the protective effect of SHP on H_2_O_2_-induced zebrafish embryos (Figure 6). The survival rate of zebrafish embryos decreased rapidly in the H_2_O_2_ (7 mmol L^−1^) environment in contrast to the control group. However, upon the addition of SHP (25, 50, 100 μg mL^−1^), the survival rate of zebrafish embryos improved with an ascending concentration of SHP (*p* < 0.01 or *p* < 0.001). These experimental findings imply that SHP has a protective effect on H_2_O_2_-induced zebrafish embryos. The influence of SHP on the H_2_O_2_-induced zebrafish heart rate was observed under the microscope (Figure 6B). The H_2_O_2_-induced heart rate in zebrafish increased significantly to 117.33% compared with the control group (*p* < 0.01). Nevertheless, after pre-treatment with SHP, the heart rate of the zebrafish decreased in a dose-dependent manner to 106.33% (*p* < 0.01). Additionally, the deformity of zebrafish was greatly improved under SHP treatment. The results showed that SHP possesses an outstanding antioxidant capacity.

### 3.9. Effects of SHP on the Activities of SOD and CAT and on the Levels of GSH-PX and MDA in Zebrafish

**Figure 7:**
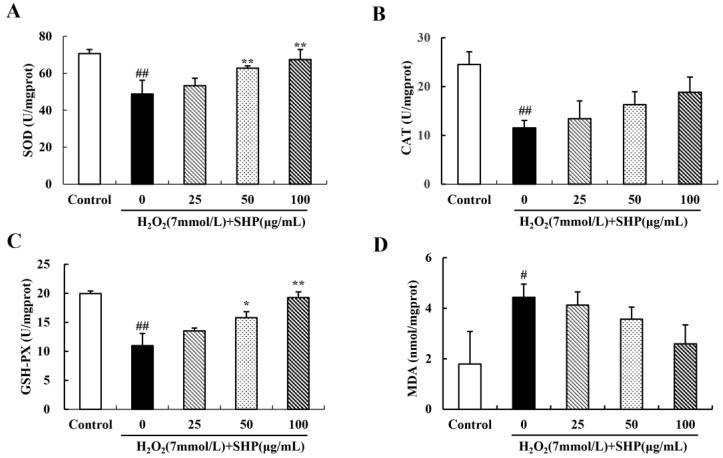
The effect of SHP on oxidative stress-related indexes of zebrafish larvae induced by H_2_O_2_ (**A**) SOD activity histogram; (**B**) CAT activity histogram; (**C**) histogram of GSH-PX content; (**D**) bar chart of MDA content. ^#^ *p* < 0.05, ^##^ *p* < 0.01 vs. control group; * *p* < 0.05, ** *p* < 0.01 vs. H_2_O_2_ model group.

To further investigate the protective mechanism of SHP against oxidative stress in zebrafish embryos, we measured the activities of SOD and CAT, as well as the contents of GSH-PX and MDA in zebrafish at 96 hpf (Figure 7). The activities of SOD and CAT and the content of GSH-PX in zebrafish in the induction group (7 mmol L^−1^ H_2_O_2_) were significantly decreased compared with the control group. However, after pretreatment with different doses of SHP, the activities of SOD and CAT and the content of GSH-PX increased in a dose-dependent manner (Figure 7A–C). The effect of SHP on MDA content in zebrafish is shown in Figure 7D. The MDA content in zebrafish in the induction group showed a significant increase, reaching 147.14%, and the difference was statistically significant compared to the control group (*p* < 0.05). Nevertheless, after pretreatment with SHP, the MDA content in zebrafish decreased in a dose-dependent manner.

### 3.10. The Role of SHP on H_2_O_2_-Induced ROS in Zebrafish

**Figure 8:**
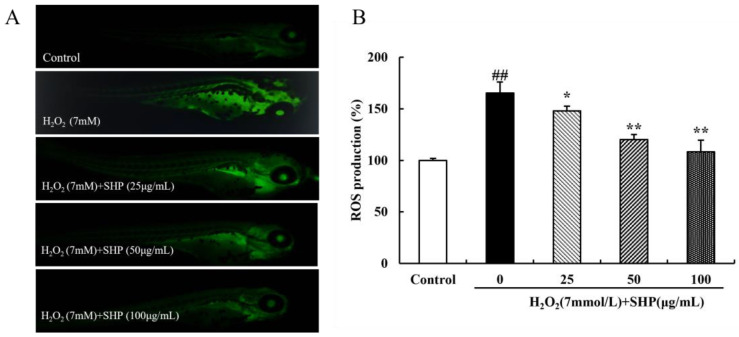
The effect of SHP on H_2_O_2_-induced ROS generation in zebrafish. (**A**) Fluorescence images of ROS expression in zebrafish; (**B**) relative ROS expression levels in zebrafish. ^##^ *p* < 0.01 vs. control group; * *p* < 0.05, ** *p* < 0.01 vs. H_2_O_2_ injury group.

The DCFH-DA probe was used to determine the ROS content in zebrafish, thereby assessing the scavenging capacity of SHP for ROS [43]. The fluorescence of zebrafish in the control group was negligible, whereas that of zebrafish in the induction group was significantly enhanced. However, the fluorescence intensity gradually declined after SHP pretreatment (Figure 8A). Similar results were shown for the relative ROS level. Compared with the control group, the ROS level in the induction group was highly expressed (165.51%), but after SHP (25, 50, 100 μg mL^−1^) pretreatment, the ROS level in the induction group decreased to 148.10%, 120.12%, 108.46% (*p* < 0.05 or *p* < 0.01) (Figure 8B).

## 4. Discussion

Oxidative and antioxidative regulatory mechanisms exist in organisms. When the organism is stimulated by the external environment, if the balance between the two is disturbed, the organism will trigger oxidative stress, which destroys the macromolecular structure of cells and ultimately results in cell apoptosis or death [44]. Previous studies have reported that the activity or content of antioxidant defense factors under oxidative stress can indirectly indicate the degree of oxidative stress in the body [41,45]. Several previous studies have also evaluated the in vitro antioxidant activity of polysaccharides from *S. horneri*. For instance, Shao et al. [22] evaluated the capacity of polysaccharides derived from *S. horneri* to neutralize free radicals. Additionally, Wen et al. [23] further clarified that polysaccharides from *S. horneri* exhibit excellent antioxidant effects by regulating gene expression levels of key enzymes involved in oxidative stress in RAW264.7 cells. We not only selected Vero cells to evaluate the in vitro antioxidant capacity of polysaccharides from *S. horneri* (SHP) but also supplemented the in vivo antioxidant activity of SHP using zebrafish as the model organism for the first time.

In this research, Vero cells were exposed to diverse concentrations of H_2_O_2_. The concentration of 0.4 mmol L^−1^ induced by H_2_O_2_ was chosen to establish an oxidative stress model, which was then utilized to investigate the antioxidant effects of SHP in vitro. The findings indicated that SHP exerted no cytotoxic impact on Vero cells and enhanced cell viability. Additionally, the ROS levels in H_2_O_2_-induced Vero cells were significantly higher compared to the control group, while ROS levels decreased in a dose-dependent manner following SHP treatment.

Under normal conditions, cells possess an endogenous antioxidant system that shields them from oxidative harm by generating antioxidant enzymes such as SOD, CAT, and GSH-PX [46,47]. SOD constitutes the most critical line of defense within the antioxidant enzyme defense system. It is capable of eliminating ROS, particularly superoxide anion free radicals, from cells, thereby enhancing the antioxidant capacity of the organism [41,48]. Our findings demonstrated that SOD activity decreased significantly in Vero cells induced by H_2_O_2_ compared to the control group, suggesting that a large number of free radicals were produced by the cells at this time and the oxidative stress was intensified. Meanwhile, the SHP treatment augmented SOD activity in a dose-dependent manner, indicating that the extent of oxidative damage in cells was mitigated.

In biological systems, lipid peroxidation is considered a toxic phenomenon that leads to a wide range of pathological conditions. Moreover, H_2_O_2_ intensifies the status of lipid peroxidation in cells [49,50]. The MDA is regarded as the ultimate decomposition product of lipid peroxidation, and its content can mirror the level of oxidative damage in cells [39]. Hence, the MDA content was determined in this study, and it was discovered that SHP reduces MDA to prevent the accumulation of free radicals in Vero cells. In summary, the antioxidant potential of SHP in protecting Vero cells from H_2_O_2_-induced oxidative stress was confirmed.

Several studies have demonstrated that oxidative stress can reduce the survival of zebrafish and result in malformation [51,52,53]. Considering the survival rate, growth, and development of zebrafish, we chose 7 mmol L^−1^ of H_2_O_2_ for zebrafish induction. Furthermore, we discovered that SHP enhances the survival of zebrafish embryos in a dose-dependent manner and mitigates zebrafish damage caused by oxidative stress. Additionally, H_2_O_2_ induction significantly increased ROS in zebrafish, but SHP treatment exhibited a dose-dependent decrease in ROS levels in zebrafish, which was also confirmed by fluorescence images under microscopy. Moreover, the enzymatic activity of SOD, CAT, and GSH-PX was significantly increased in zebrafish, and the MDA content was effectively suppressed, thereby protecting zebrafish from H_2_O_2_-induced oxidative damage. In conclusion, firstly, SHP might lower ROS levels and eliminate oxidative free radicals by enhancing the enzymatic activity of the oxidative defense system, thereby reducing the oxidative stress response of the organism. Secondly, the sulfates and uronic acids present in polysaccharides also enhance hydrogen donation and thus exert antioxidation activities to convert ROS into inactive or stable compounds [42]. Thus, polysaccharides are indispensable contributors to maintaining the radical scavenging system.

## 5. Conclusions

SHP derived from *S. horneri* can effectively safeguard the vitality of Vero cells, enhance the activity of the SOD enzyme, lower the content of MDA, eliminate intracellular ROS, and mitigate the oxidative damage caused by H_2_O_2_. Meanwhile, SHP can enhance the activities of SOD, CAT, and GSH-PX enzymes in zebrafish, effectively suppressing the levels of MDA and ROS, thereby reducing the oxidative damage caused by H_2_O_2_ and exerting a protective effect on zebrafish. Utilizing zebrafish as a model organism, we present, for the first time, a detailed demonstration of the antioxidant effects of SHP in vivo. This study validates the potential of SHP as a natural antioxidant to augment the protective effects against oxidative stress and to be considered for applications in the biomedical, cosmetic, and functional food industries.

## Figures and Tables

**Table 1:** The effect of SHP on SOD activity and MDA content in Vero cells.

Sample	(μg/mL)	Vero	
		SOD (U/mgprot)	MDA (nmol/mgprot)
Control	-	52.43 ± 1.31	7.17 ± 1.29
H_2_O_2_	-	22.9 ± 4.46 ^##^	19.04 ± 2.53 ^##^
SHP	25	28.28 ± 5.77	14.09 ± 0.51 **
	50	30.80 ± 6.04	11.09 ± 2.10 **
	100	40.70 ± 8.95 **	9.39 ± 0.92 **

Note: ^##^ *p* < 0.01 (control group vs. H_2_O_2_ injury group); ** *p* < 0.01 (SHP vs. H_2_O_2_ injury group).

## Data Availability

The datasets generated during and/or analyzed during the current study are available from the corresponding author on reasonable request.
